# Medicina tradicional como tratamiento de la COVID-19 en estudiantes y familiares en una universidad de la sierra del Perú

**DOI:** 10.1016/j.aprim.2022.102526

**Published:** 2022-11-14

**Authors:** Gladys Bernardita León Montoya, Mercedes Acosta Román, María Esther Saavedra Chinchayán, Soledad Almonacid Quispe

**Affiliations:** a Universidad Nacional Toribio Rodríguez de Mendoza de Amazonas, Chachapoyas, Amazonas, Perú; b Universidad Nacional Autónoma de Tayacaja Daniel Hernández Morillo Perú, Huancavelica, Perú

La pandemia por SARS-CoV-2, que provoca la COVID-19, ha puesto en jaque algunos de los sistemas de salud del mundo, debido a que no se encontraban preparados para una pandemia viral de esta naturaleza. Por ahora no existe un tratamiento definitivo contra este virus, razón por la cual la mayoría de países del mundo han hecho uso también de su medicina tradicional o complementaria. Los asiáticos están usando la medicina ancestral china, y en Perú también, al tener comunidades alto andinas, se está haciendo uso de las plantas medicinales y de otras alternativas, cuyas propiedades se conocen desde siempre y su uso está legislado en la Ley No. 27811 que establece un régimen especial de protección de los conocimientos colectivos de los pueblos indígenas vinculados a los recursos biológicos, donde también se contemplan las plantas medicinales como parte de la cosmovisión^1^. Tayacaja es una provincia que pertenece a Huancavelica y que está ubicada en el centro sur del Perú, donde el 65,23% de la población de 5 o más años de edad refiere que el idioma o lengua materna es el quechua^2^; es decir, es una población quechua hablante. Pampas pertenece a Tayacaja, por lo tanto se encuentra en este contexto y es donde está ubicada la universidad; los pobladores de esta ciudad también hacen uso de la medicina tradicional, motivo por el cual la presente investigación tuvo como objetivo conocer el uso de la medicina tradicional en estudiantes y/o familiares que tuvieron COVID-19 de la Universidad Nacional Autónoma de Tayacaja Daniel Hernández Morillo. Esta investigación se realizó durante los meses de mayo y junio de 2022 y la población estuvo constituida por 518 estudiantes de las 5 carreras profesionales que tiene la universidad. Se trata de un estudio cuantitativo, descriptivo, transversal y retrospectivo, en el que se utilizó el muestreo no probabilístico por conveniencia. Se obtuvo una muestra de 271 estudiantes que se habían infectado con el SARS-CoV-2 y que utilizaron medicina tradicional. Así mismo, estos estudiantes dieron información de sus familiares que habían tenido COVID-19, y que al igual que ellos utilizaron la medicina alternativa, haciendo un total de 252 familiares. A los estudiantes se les aplicó —previa aceptación del consentimiento informado— una encuesta *ad hoc* a través de Google forms, la cual fue elaborada por las autoras y validada por 3 expertos. Los resultados encontrados fueron que el 99,63% de los estudiantes con COVID-19 y el 89,0% de los familiares con COVID-19 utilizaron el uso de las plantas medicinales para tratarse contra la COVID-19, siendo las plantas más utilizadas en ambos grupos en primer lugar el eucalipto seguido del matico, utilizando las hojas de ambas plantas. La forma de preparación que más usaron ambos grupos fue la infusión, seguida del jarabe y las pociones. La mayoría lo utilizó de forma oral y en baños para los estudiantes y para los familiares de manera oral y por frotación. Asimismo se encontró que el uso de animales, minerales y prácticas espirituales en ambos grupos fueron utilizados por una minoría, lo que nos lleva a concluir que en este caso tanto los estudiantes como los familiares se centraron en el uso de la fitoterapia y no en el resto de alternativas.

## Consideraciones éticas

Se cuenta con el consentimiento informado de los sujetos de investigación.

## Financiación

La presente investigación no ha recibido ayudas específicas provenientes de agencias del sector público, sector comercial o entidades sin ánimo de lucro.

## Conflicto de intereses

Se declara no tener conflicto de intereses.
